# Rice Husk Biochar: A Promising Material for the Development of Electrochemical Devices for Tartrazine Determination

**DOI:** 10.1021/acsomega.5c12503

**Published:** 2026-08-10

**Authors:** Leandro Silva de Almeida, Ana Paula de Oliveira Lopes Antunes, Eliézer Quadro Oreste, Mery Luiza Garcia Vieira, Luiz Antonio de Almeida Pinto, Tito Roberto Sant’Anna Cadaval, Daiane Dias

**Affiliations:** † Laboratório de Eletroespectro Analítica (LEEA), School of Chemistry and Food, Federal University of Rio Grande, Rio Grande 96203-900, Brazil; ‡ Laboratório de Tecnologia Industrial, School of Chemistry and Food, Federal University of Rio Grande, Rio Grande 96203-900, Brazil

## Abstract

This study presents the successful application of rice husk biochar (BC) in modifying carbon paste electrodes for the voltammetric determination of tartrazine (Tz) in commercially available juices. The BC was produced under different pyrolysis conditions using a central composite rotational design, in which the evaluated parameters were heating rate, biomass amount, temperature, and time. All BC samples obtained were used to modify the carbon paste electrode, and the best pyrolysis conditions (400 °C, 20 g of rice husk, a heating rate of 25 °C min^–1^, and a pyrolysis time of 180 min) were determined based on the highest Tz current response obtained by differential pulse voltammetry. The obtained material was characterized by thermogravimetry, N_2_ adsorption/desorption isotherms, scanning electron microscopy, Fourier transform infrared spectroscopy, and electrochemical impedance spectroscopy. The electrode composition was also evaluated, and the highest current response Tz was obtained in the electrode containing 70% graphite, 22.5% liquid paraffin, and 7.5% BC. The influence of experimental parameters was then evaluated for this electrode, and the best conditions were obtained in 0.1 mol L^–1^ phosphate buffer solution (pH = 2), *E*_step_ of 0.008 V, and amplitude of 0.12 V. The Tz analytical curve was linear from 50 to 350 mg L^–1^, and the detection and quantification limits calculated were 0.17 and 1.57 mg L^–1^, respectively. To evaluate accuracy, recovery tests were used, yielding values between 84 and 117%. The method was successfully applied to six samples of two juice brands in the market, demonstrating its practicality and reliability. These results highlight the potential of BC-based electrodes as sustainable and effective alternatives for food analysis.

## Graphical abstract

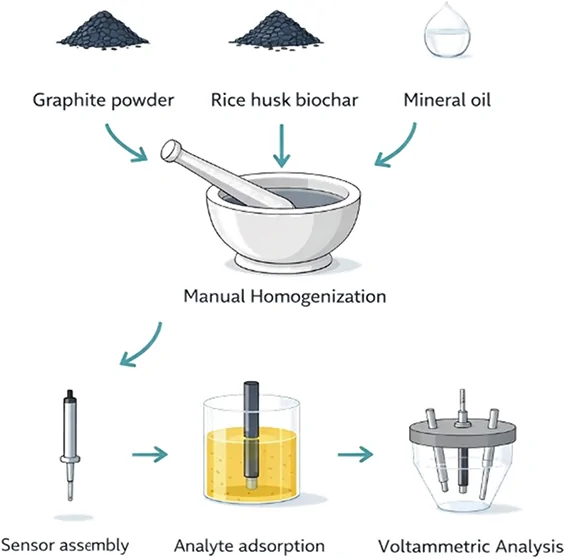

## 1 Introduction

Voltammetry has been widely used to determine organic compounds using a classic mercury electrode.^1^ However, the use of mercury-based electrodes has become less common in recent years due to their toxicity and environmental concerns, particularly in routine analytical and food analysis applications. As a result, considerable efforts have been devoted to the development of alternative electroanalytical sensors using nontoxic materials derived from renewable and low-cost sources.^2–4^ In this sense, electroanalytical methods have been constantly applied in the determination of a wide variety of analytes (toxic metals, pharmaceutical compounds, food additives, pesticides, and others)^5–7^ through the construction of modified sensors based on carbon nanomaterials, metallic nanoparticles, conductive polymers, and hybrid composites.^8,9^

Among these materials, BC deserves special attention because it can be produced from agricultural residues such as corn, sugar cane, coconut, wheat, cassava, and cotton, among others.^10^ Rice husk is a type of biomass that has already been explored to produce products such as ash, BC, and activated carbon, which can be applied in environmental remediation, energy storage, catalysis, and electrochemical sensing.^11–14^ According to data from the FAO (Food and Agriculture Organization of the United Nations), world rice production in 2025/2026 was about 563.4 million tons.^15^ Brazil is the tenth largest rice producer globally, accounting for around 1.6% of global production.^15^ This fact demonstrates the urgency of adequate environmental alternatives and studies aimed at taking advantage of abundant biomass.^16^

Considering the limitations associated with conventional electrode materials, the development of chemically modified carbon paste electrodes (CMPE) from BC has emerged as a promising alternative, not only because it is safe and environmentally friendly but also due to the intrinsic properties of BC, such as high electrical conductivity, chemical stability, large specific surface area, well-developed porosity, and compatibility with various electrolyte systems, which enhance the electrocatalytic response of the electrodes.^2,16–18^ However, these properties can be enhanced depending on the parameters used in the thermal degradation process to obtain BC, and this step is crucial for careful studies.^19,20^ The experimental design methodology can be used to perform this kind of study by evaluating combinations of pyrolysis parameters and thereby help obtain an ideal composite for developing electrodes.

Electroanalytical determinations of dyes in food are currently gaining ground compared to traditional techniques, such as chromatography.^21^ Thus, due to the presence of chromophoric and auxochromic groups, synthetic dyes have organic structures characterized by chromophores and auxochromes, and many of them, such as Tz, have the azo group (−N = N−) that can present a risk to human health, since aromatic amines are considered carcinogenic.^22,23^ For this reason, authorities responsible for food safety determine the maximum concentrations of dyes in food. There is a relevant concern regarding the dye Tz, as it is associated with allergies and hypersensitivity.^24^ Although several BC-based electrochemical sensors have been reported in the literature, most studies focus on activated or chemically modified BCs or on complex nanostructured materials. In contrast, the use of rice husk-derived BC obtained under systematically optimized pyrolysis conditions and directly applied as a modifier in carbon paste electrodes (CPE) for Tz determination remains scarcely explored.

In this scenario, the use of BC derived from rice husk may represent a promising alternative material for electroanalytical applications. To our knowledge, BC derived from rice husk has not yet been used to produce voltammetric electrodes, despite its great potential, mainly due to its high adsorptive capability and large surface area.^11^ Therefore, this study aimed to develop and characterize electrodes modified with BC from rice husk and apply them to determine Tz dye in commercially available juices by differential pulse voltammetry (DPV). In this context, the novelty of this work lies in (i) the use of rice husk BC as a sustainable modifier for CPEs, (ii) the systematic optimization of pyrolysis parameters using experimental design, and (iii) the development of a simple and low-cost electroanalytical method with practical applicability in real samples.

## 2 Materials and Methods

### 2.1 Chemicals and Reagents

Rice husks were provided by a local industry in Rio Grande, Rio Grande do Sul, Brazil, during the fall of 2021. The material was washed with distilled water to remove adhered impurities and dried in an oven at 100 °C for 24 h. Afterward, grinding and sieving were performed, and particles smaller than 425 μm were used. The solutions used as electrolytes in the voltammetric cells were obtained from phosphate buffer solution (pH = 1.5, pH = 2.0, pH = 3.0, pH = 4.0, and pH = 5.0) 0.1 mol L^–1^ by diluting an adequate volume of concentrated H_3_PO_4_ (Acros, Brazil). The pH values were adjusted with a 5.0 mol L^–1^ sodium hydroxide solution (Synth, Brazil). For the electrochemical impedance spectroscopy (EIS) experiments, 1.0 × 10^–3^ mol L^–1^ of a K_4_Fe­(CN)_6_ (Synth, Brazil) solution was added to a 0.1 mol L^–1^ KCl (Synth, Brazil) solution. The glassware used to determine the Tz dye was decontaminated by immersion in a 10% (v/v) HNO_3_ solution (Synth, Brazil) for 24 h. The voltammetric cells were also decontaminated by immersion in a 20% (v/v) HNO_3_ solution (Synth, Brazil). After decontamination, the glassware and the voltammetric cells were washed with ultrapure water.

### 2.2 Experimental Design for Rice Husk BC Production

The influences of the pyrolysis factors were evaluated using a central composite rotational design (CCRD). The number of experiments was defined by 2*^n^* + 2*n* + *C*, in which *C* = 3 (represents the central points) and *n* = 4 (represents the number of independent variables). The parameters studied were heating rate (5–25 °C min^–1^), biomass amount (10–50 g), temperature (200–600 °C), and time (60–180 min). The planning resulted in 27 rice husk pyrolysis experiments, which were randomly oriented. The analyses were arranged in the order specified in the Windows version of the Statistics software, version 8.0.

### 2.3 Preparation of CMPE with BC

This step prepared 27 electrodes modified with BC pyrolyzed under different conditions and one electrode without modification. Previously, the CMPE was obtained by manually mixing 70% graphite powder (nonanalytical), 5% BC, and 25% mineral oil for 40 min. The paste was packaged in syringes (6 mm in diameter) with a copper rod as the electrical contact. Afterward, the devices obtained were submitted to preliminary adsorption tests using a Tz dye solution (0.15 mg L^–1^) with solution stirring for 1 min under open-circuit conditions (without applying a potential). Next, the device was washed with ultrapure water and placed in a voltammetric cell containing 10 mL of 0.1 mol L^–1^ phosphate buffer solution (pH = 3.0). Scans were performed by DPV from 0.5 to 1.2 V with an *E*_step_ of 0.003 V and an amplitude of 20 mV.

The response evaluated for each device (27 CMPE with BC and one without modification) was the current intensity Tz determinations (*n* = 5). Based on the highest current responses shown on the response surfaces, another combination of pyrolysis parameters was proposed (400 °C, 20 g of rice husk, a heating rate of 25 °C min^–1^, and a pyrolysis time of 180 min) and a new electrode, named CMPE-BC28 was obtained by mixing 70% graphite powder (nonanalytical), 5% BC, and 25% mineral oil for 40 min manually, that was deeply characterized and applied.

### 2.4 Evaluation of Experimental Parameters

For the choice of BC proportion, CMPEs were prepared with different ratios of modifiers (2.5, 5.0, 7.5, and 10%), and the Tz response was evaluated (*n* = 5) under the same conditions described in Section 2.3. Then, parameters such as pH (1.5–5), adsorption time (20–80 s) of Tz (0.15 mg L^–1^) in an open circuit under agitation, *E*_step_ (0.002–0.012 V), and amplitude (0.06–0.14 V) were also evaluated.

### 2.5 Evaluation of the Figure of Merit

After optimization of the experimental parameters, all figures of merit were evaluated using CMPE-BC28 (analysis by DPV) in 10 mL of phosphate buffer solution 0.1 mol L^–1^ (pH = 2), *E*_inicial_: 0.5 V; *E*_final_: 1.2 V; amplitude: 0.12 V, and *E*_step_: 0.008 V. Method linearity was evaluated over the range of 50 to 350 mg L^–1^. The limits of detection (LOD) and quantification (LOQ) were calculated using the ratios 3 × (σb/m) and 10 × (σb/m), respectively, where σb is the standard deviation obtained for ten blank measurements and m is the analytical sensitivity (angular coefficient). For the accuracy evaluation, addition and recovery experiments were performed. All experiments were performed in replicate (n = 5).

### 2.6 Tz Determinations in Powdered Juices

Tz was determined in six samples of commercially available juices (orange, lemon, and passion fruit flavored) of two brands classified as brands 1 and 2. 0.5 g of the samples was dissolved in 10 mL of ultrapure water, followed by adsorption on the CMPE-BC28 for 60 s. Afterward, the electrode was washed with ultrapure water and placed in the voltammetric system for analysis under the same conditions described in Section 2.5.

### 2.7 Kinetic Behavior in the CMPE-BC28

Cyclic voltammetry experiments were performed (0.5 to 1.4 V) at scan rates from 200 to 1000 mV s^–1^ to evaluate the electrochemical kinetic behavior of oxidation processes on the surface of the modified electrode under the same conditions described in Section 2.5.

### 2.8 Electrochemical Characterization of the CMPE-BC28

The electrochemical characterization of the CMPE-BC28 and CPE (without modification) was carried out by EIS using 1.0 × 10^–3^ mol L^–1^ K_4_Fe­(CN)_6_ solution added in 0.1 mol L^–1^ KCl solution as a redox probe. The electrochemical parameters were amplitude of 10 mV, frequency range of 100 kHz to 100 mHz, and half-wave potential (*E*_1/2_) of [Fe­(CN)_6_]^3–^/[Fe­(CN)_6_]^4–^. The Randles equivalent electric circuit was used to fit the spectra, allowing the solution resistance values (*R*_s_) and the charge transfer resistance (*R*_tc_).

## 3 Results and Discussion

### 3.1 Preliminary Evaluation of the Tz Voltammetric Response in Electrodes Modified with BC (CMPE-BC)

A preliminary study evaluated which electrode presented the highest response to Tz determination, as depicted in Figure 1 (see the data in Table S1).

**Figure 1:**
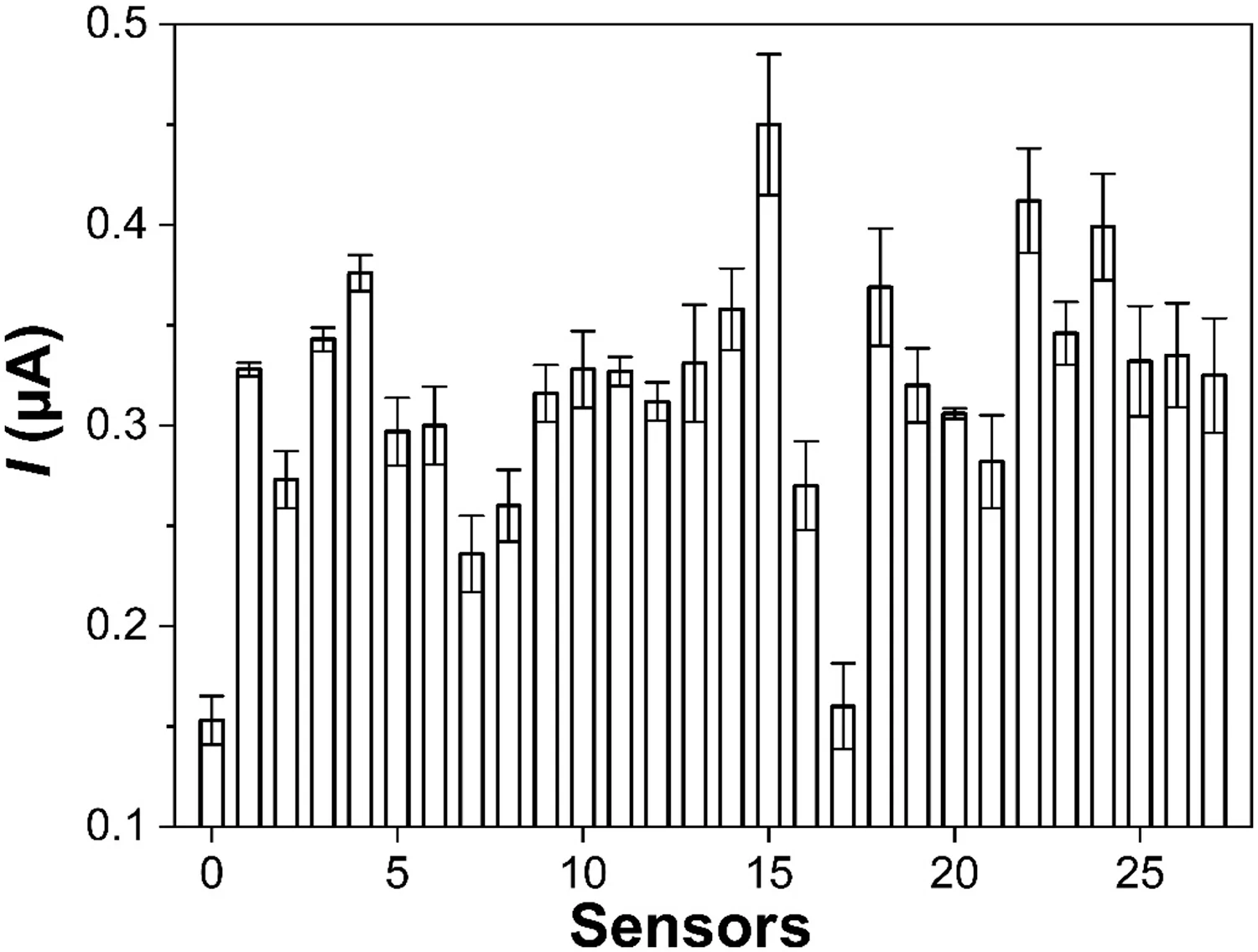
Current responses of Tz (open-circuit adsorption of Tz 0.15 mg L^–1^ during 1 min) in the modified electrodes by DPV. Experimental conditions: phosphate buffer solution 0.1 mol L^–1^, pH = 3.0, *E*_initial_: 0.5 V, *E*_final_: 1.2 V, *E*_step_: 0.003 V, amplitude: 20 mV.

As depicted in Figure 1, the Tz response in CPE without modification (electrode 0) is smaller than almost all CMPE. In the first part of this study (up to electrode 27), the highest current responses were obtained in CMPE-BC15, CMPE-BC22, and CMPE-BC24. Considering that the BC used in these modifications was derived from rice husks pyrolyzed at 300 and 400 °C, this behavior must be related to the beginning of cellulose depolymerization at this temperature.^25^ Additionally, in this carbonaceous matrix, the amorphous structure predominates and tends to form sheets of polyaromatic graphene, improving the adsorption capacity.^25,26^ In contrast, the CMPE-BC17 electrode from rice husks pyrolyzed at the lowest temperature studied (200 °C) had the lowest current response (0.16 μA). This is due to incomplete degradation of the carbonaceous material.

Further evaluation of the electrode responses was carried out using the surface response. Figure S1 shows the influence of time and temperature on the current values of Tz. As observed at 500 °C, current responses tended to be higher with a shorter pyrolysis time (60 min). This fact can be explained by the fact that, in shorter pyrolysis times at high temperatures, a portion of partially aromatized carbons is still present in the material, in addition to some residual oxygenated functions, which tend to favor the adsorption of Tz.^27^ Concomitantly, it is understood that the surface of the material is not completely degraded at this stage, preserving micropores that facilitate the diffusion of the analyte, consequently generating higher observable currents. However, at temperatures below 500 °C, a longer pyrolysis time was required to maintain the current intensity at the same level. The results shown in Figure S2 suggest the improvement of electrode capacity using 20 to 40 g of rice husk at 180 min of pyrolysis. The influence of the heating rate on the variables mass, temperature, and pyrolysis time is shown in Figures S3–S5, respectively. It was observed that masses of 10 to 20 g of rice husk require a heating rate of 25 °C min^–1^ to get BC with a higher current response of Tz. In comparison, masses around 50 g tend to improve material properties at slower heating rates, such as 5 °C min ^–1^. Regarding the pyrolysis temperature in Figure S3, it can be inferred that between 400 and 500 °C allows a good electrochemical response of BC, regardless of the heating rate. A tendency to improve the material response by combining a longer time (180 min) and a heating rate of 25 °C min^–1^ was also observed (Figure S4). However, Figure S6 shows that for biomass pyrolyzed at 400 to 500 °C over 20 to 40 g, the current responses of the electrode tend to be similar.

Considering these results, a new combination of pyrolysis parameters was proposed using 400 °C, 20 g of rice husk, a heating rate of 25 °C min^–1^, and a pyrolysis time of 180 min. The obtained BC was used to modify the electrode (named CMPE-BC28). As depicted in Figure 1, the current response of Tz in this electrode (0.45 μA) was around 82% higher than the CPE without modification (0.08 μA). Therefore, this electrode was chosen for the following experiments.

### 3.2 Optimization of Experimental Parameters

As depicted in Figure 2a, the proportion of BC in electrode modification influenced the current responses of Tz. Among the proportions evaluated, the highest current values were obtained using 7.5%. Using the proportions above (like 10%), it is possible to observe a decrease in the current intensity, and similar behavior was observed by Kalinke and co-workers, indicating that the BC interfered with the electroactive capacity of the graphite.^28^ From the data obtained, the experiments in the sequence were carried out using 7.5% BC as a modifier in the carbon paste composites.

**Figure 2:**
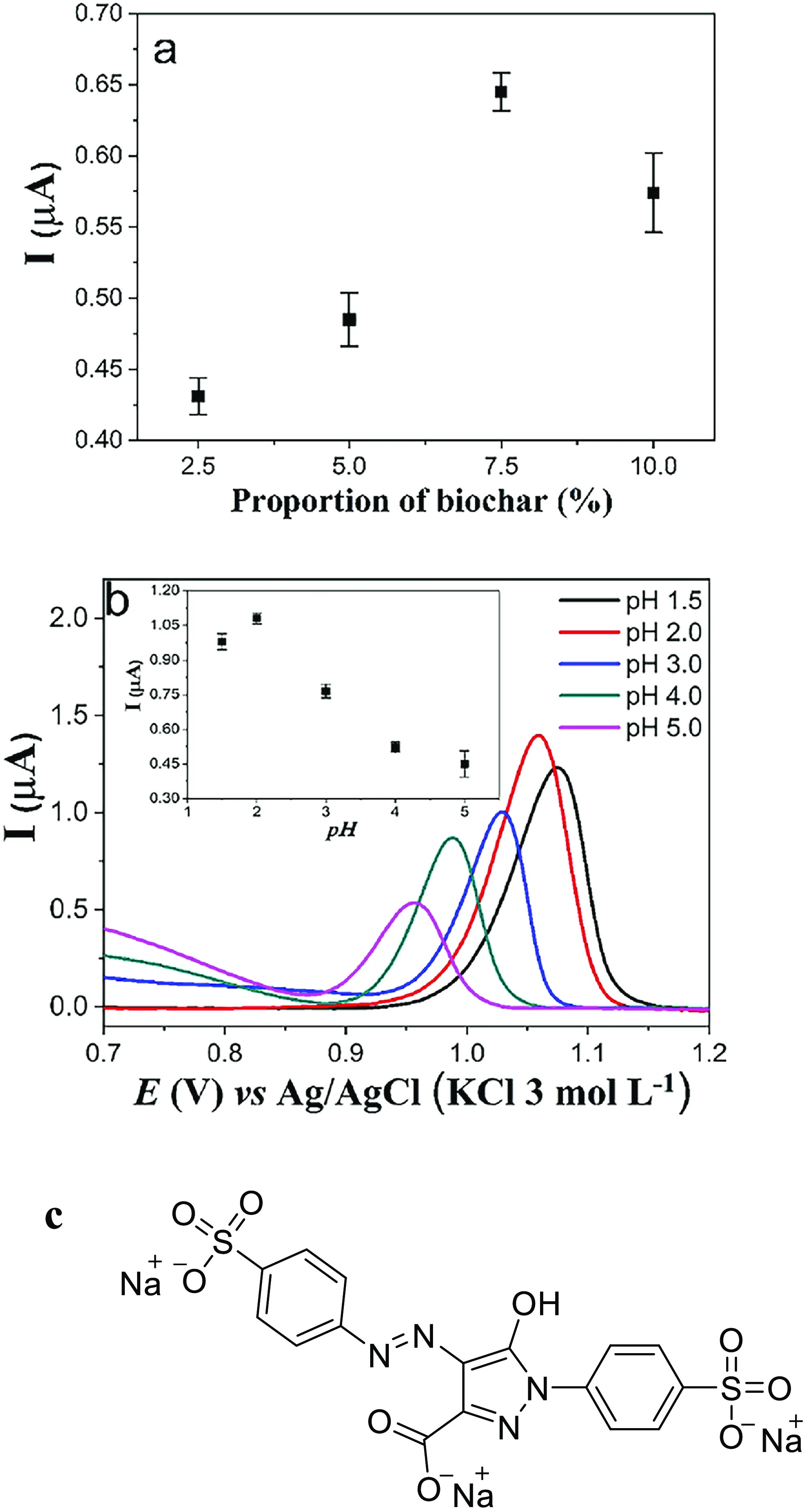
Effect of (a) BC proportion (from 2.5 to 10%) and (b) pH on the voltammogram and current responses (inset) of Tz (open-circuit adsorption of Tz 0.15 mg L^–1^ during 60 s) in the CMPE-BC28 by DPV. Experimental conditions: phosphate buffer solution 0.1 mol L^–1^, pH = 3.0, *E*_initial_: 0.5 V, *E*_final_: 1.2 V, *E*_step_: 0.003 V, amplitude: 20 mV. (c) Chemical structure representation of Tz.

Based on the work published by Karimi and co-workers, phosphate buffer solution was chosen as the electrolyte, as Tz oxidation tends to be more sensitive in this electrolyte.^29^ As can be seen in Figure 2b, there was an increase in peak current intensity with the rise in hydrogen concentrations (up to pH = 2.0) as well as a shift of Tz oxidation potential to a more negative direction, which is the phenomenon mentioned in the literature.^29^ This behavior can be associated with the molecular structure of Tz (Figure 2c), which contains azo (−N = N−) and sulfonate (−SO_3_^–^) groups, which influence the protonation state and also the electron density distribution of the molecule. These conditions facilitate electron transfer coupled with proton transfer.^23^ Additionally, the slope obtained between the peak oxidation potentials and the pH values was near (0.0479 V), the theoretical value of 0.059 V, indicating that an equal number of protons and electrons are involved in the Tz oxidation process.^29,30^ This result is consistent with a proton–electron coupled transfer (PCET) mechanism, in which the oxidation process involves the simultaneous transfer of electrons and protons, as expected for azo compounds in an acidic medium. Under these conditions, protonation of the azo group facilitates the formation of a cation-type radical intermediate, stabilized by resonance along the conjugated aromatic system.^29,30^ Once the highest current response of Tz was obtained at pH = 2.0, this value was chosen for the work sequence. Furthermore, the slight deviation from the theoretical Nernst slope can be attributed to secondary effects, such as adsorption at the electrode surface and interactions with functional groups present in the biochar, which may influence the overall electrochemical response. Additionally, ANOVA was performed to evaluate the effects of biochar proportion and pH at a 99.5% confidence level, and significant statistical variations were observed, which means that both variations in the proportion of biochar and pH significantly influence the current response under the conditions studied.

The influence of the adsorption time was also evaluated, as depicted in Figure S7. The current response to Tz increased by up to 60 s. However, due to saturation of the BC’s active sites, the current response decreased by 80 s, which resulted in interference in the current responses.^26^ Therefore, 60 s were chosen for the following experiments.

As shown in Figure S8, the highest *E*_step_ values generally improve the Tz current intensities (up to 0.8 mV). On the basis of the Tukey test, there was no statistically significant difference between the current obtained using 0.008 and 0.010 V (confidence level of 95%). However, given the lowest standard deviation, the *E*_step_ value of 0.008 V was fixed for the following experiments.

As shown in Figure S9, with an increase in amplitude, the current intensity increased. Once there was no statistically significant difference between the current values obtained at 0.12 and 0.14 V (95% confidence level), 0.12 V was chosen, since it presented a lower standard deviation between replicates.

### 3.3 Physical–Chemical Characterization of Biomass, BC, and Modified Electrode

Figure 3a shows the results of the thermogravimetric characterization of the rice husk, where mass loss events associated with the decomposition of the lignocellulosic structure were observed based on the first derivative of thermogravimetric analysis. The first observed peak (between 50 and 100 °C) is related to water loss. In the second peak (between 300 and 400 °C), there is a predominance of sequential degradation of hemicellulose and cellulose.^31^

**Figure 3:**
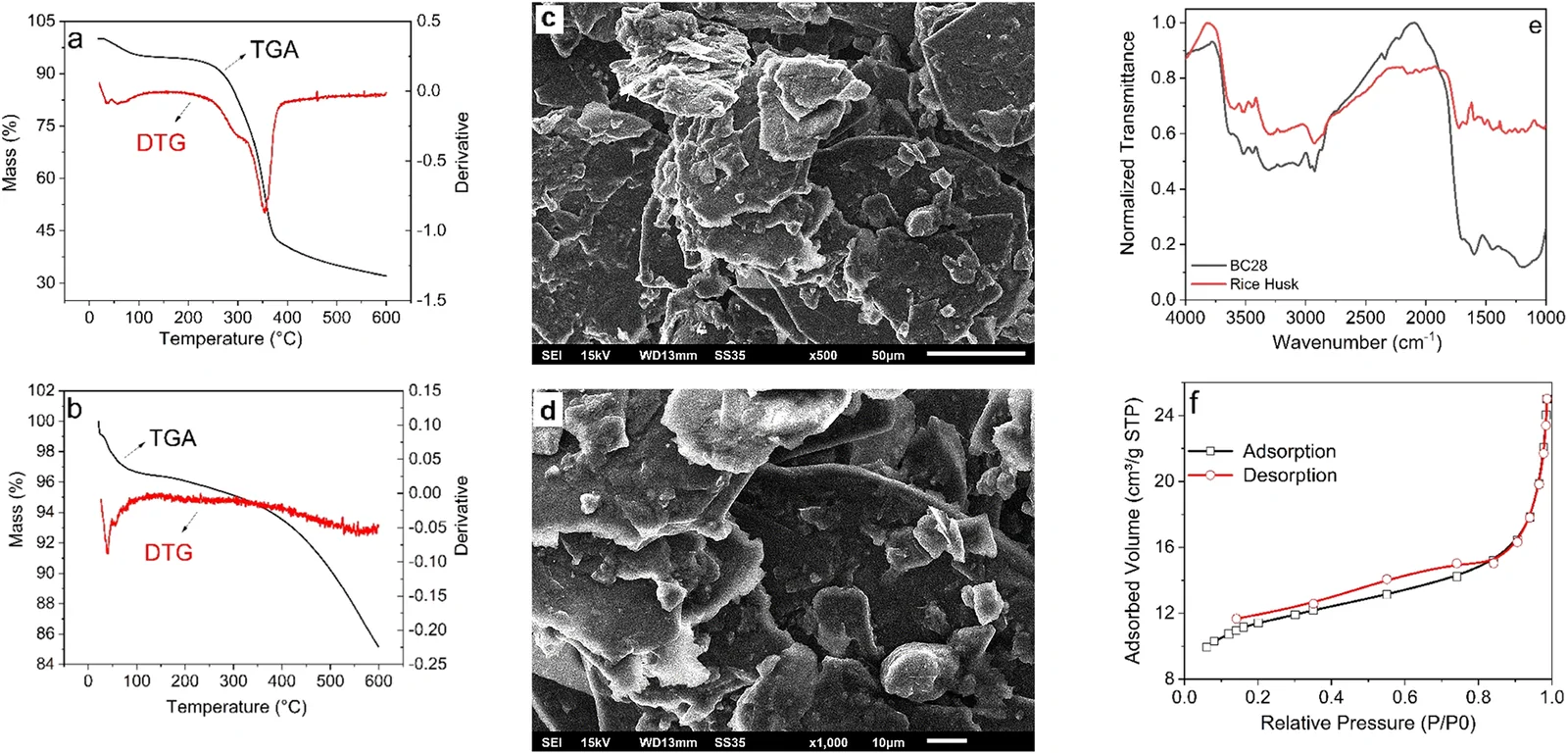
Physical–chemical characterization and thermogravimetry of the rice husk (a) and BC (b); scanning electron microscopy images of BC28 with 500× (c) and 1000× magnification (d); FTIR spectra of rice husk and BC28 (e); and N_2_ adsorption/desorption isotherms of BC28 (f).

Figure 3f shows the N_2_ adsorption/desorption isotherms obtained by the Brunauer–Emmett–Teller (BET) method for determining the textural characteristics of BC28. The BC28 showed a surface area of 35.939 m^2^ g^–1^, a total pore volume of 0.037 cm^3^ g^–1^, and a pore diameter of 4.13 × 10^–3^ μm, indicating the predominance of mesoporous materials.^32^ In the scanning electron microscopy (SEM) images of BC28 shown in Figure 3c,d, it can be observed that the material has a rough surface and irregular cavities. This morphology has been attributed to pyrolysis products formed between 300 and 400 °C.^28^ Additionally, Figure 3e shows the functional groups present in the structure of rice husk and BC28 obtained by Fourier transform infrared spectroscopy (FTIR), where the bands from 1600 to 1750 cm^–1^ represent groups related to carboxyl and carbonyls, such as carboxylic acids, ketones, and esters formed during pyrolysis.^33^ Bands below 1080 cm^–1^ indicate the presence of groups containing silicon oxides.^34^ Oxygenated groups related to hemicellulose and cellulose are shown in spectrum bands from 1000 to 1200 cm^–1^ and from 1200 to 1400 cm^–1^.^33^ Verifying an agreement between the results obtained by thermogravimetric analysis and FTIR was possible. Differences in band intensities between rice husk and BC28 indicate the influence of thermal degradation. In the FTIR spectra, the bands exhibit a similar profile in both samples. This means that pyrolysis at 400 °C results in incomplete degradation because the remaining carbonic groups contain oxygen.

### 3.4 Kinetic Evaluation of Tz Behavior in CMPE-BC28

As shown in Figure S10, the Tz showed a current response only in the anodic direction (at 1.05 V) on the CMPE-BC28, indicating an irreversible process.^35^ Additionally, an electrochemical process involving diffusional and adsorptive contributions is indicated by Figure 4a,b. Furthermore, Figure 4c, representing a graph of log­(*I*_pa_) as a function of log­(*v*), presents a slope of 0.9167 (*R* ^2^ = 0.987), in accordance with the equation log­(*I*_pa_) = 0.9167 log­(*v*) −8.6258. Thus, when considering the theoretical slope values of 0.5 for a diffusion-controlled process and 1.0 for an adsorptive process,^30^ a mixed process is supported, with a predominance of an adsorption process.

**Figure 4:**
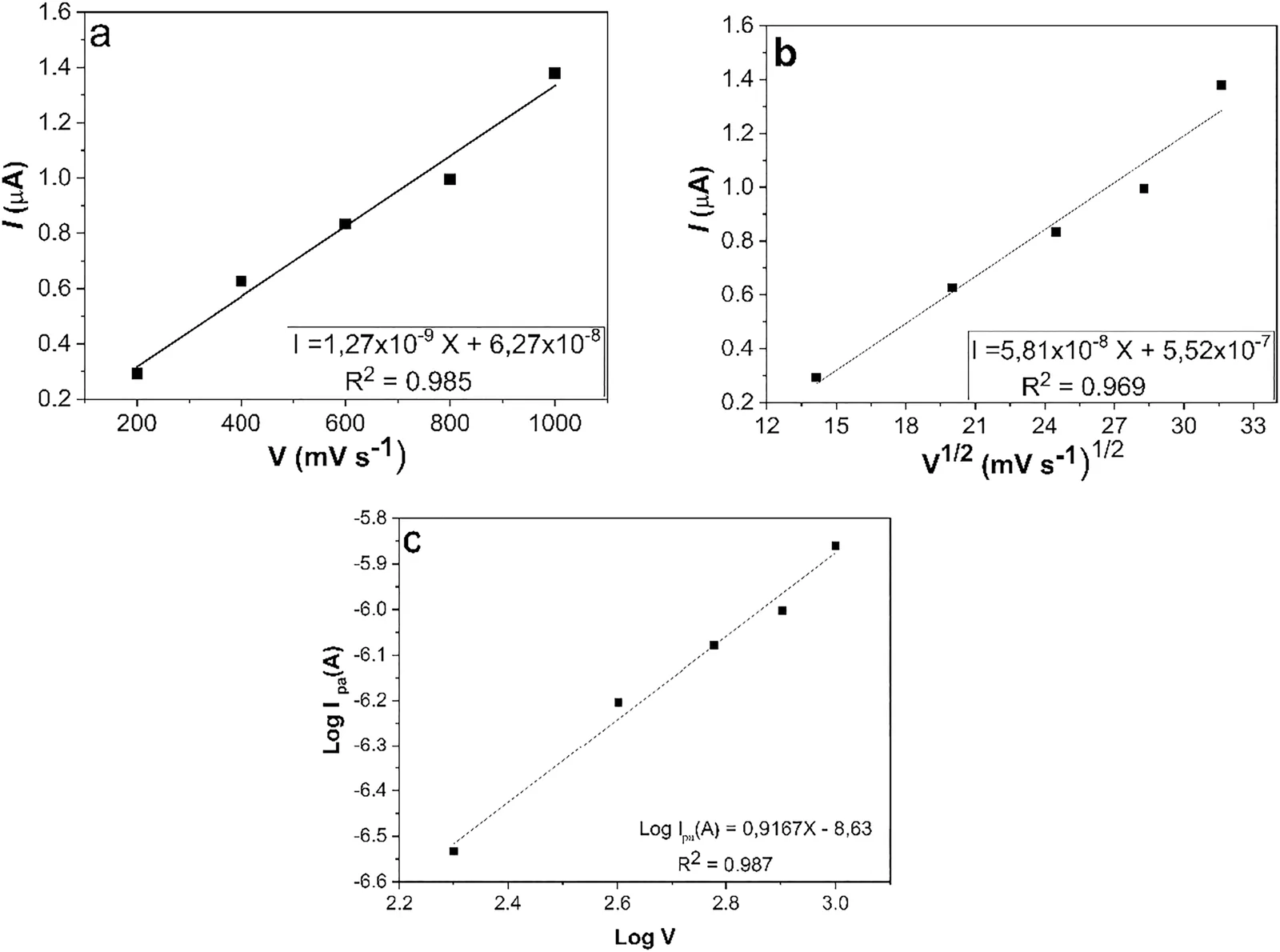
Relationship between (a) *i* and v, (b) *i* and v^1/2^, and (c) log *i* and log v of Tz (open-circuit adsorption of 0.5 g L^–1^ during 60 s) on the CMPE-BC28 from 200 to 1000 mV s^–1^. Experimental conditions: phosphate buffer solution 0.1 mol L^–1^, pH = 3.0, *E*_initial_: 0.5 V, *E*_final_: 1.4 V, *E*_step_: 0.003 V.

### 3.5 Characterization of CPE and CMPE-BC28 by EIS

The Nyquist plots shown in Figure 5a exhibit a semicircle in the high-frequency region, corresponding to the Faradaic processes of the electrical double layer. The linear region refers to low-frequency phenomena arising from semi-infinite diffusion processes.^30^

**Figure 5:**
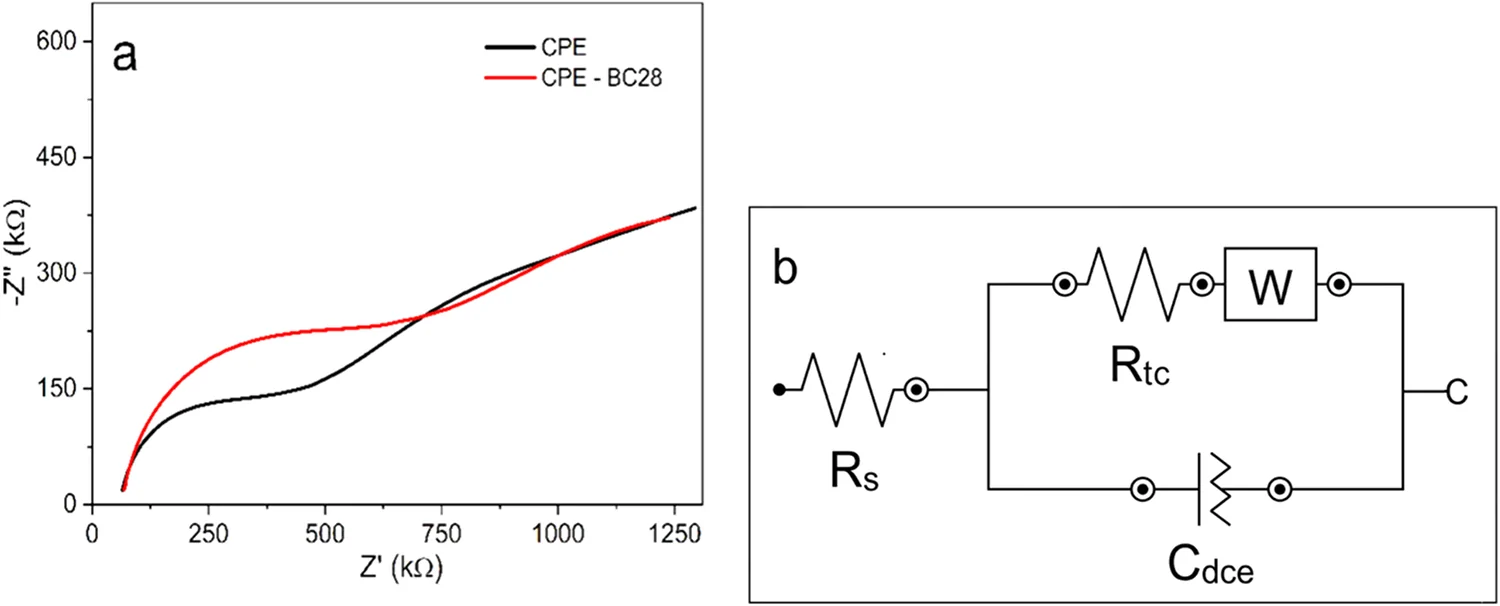
(a) Nyquist diagram recorded in 0.1 mol L^–1^ KCl containing 1.0 × 10^–3^ mol L^–1^ [Fe­(CN)_6_]^3–^/[Fe­(CN)_6_]^4–^ at CPE (black) and CMPE-BC28 (red); (b) corresponding equivalent electrical circuit.

To fit the experimental EIS data, the Randles equivalent circuit was used, represented in Figure 5b, with the charge transfer resistance (*R*_tc_), referring to the redox processes of [Fe­(CN)_6_]^3–^/[Fe­(CN)_6_]^4–^, being in series with the Warburg (W), which represents the diffusional process at low frequencies. Both are parallel to the electrical double-layer capacitance (*C*_dce_) capacitor. This entire set is in series with the resistance of the solution (*R*_s_).^36^ Comparison between Nyquist diagrams allows inferring that the presence of BC in the CMPE-BC28 composite has increased the *R*_tc_ values compared to the bare CPE (*R*_tc_ of 505.70 and 831.61 Ω for CPE and CMPE-BC28, respectively). Although a higher Rct typically indicates slower electron transfer kinetics, the modified electrode exhibits higher sensitivity to Tz. This apparent paradox can be explained by adsorption-controlled processes. The biochar introduces a porous structure and functional groups that enhance Tz accumulation at the electrode surface, effectively preconcentrating the analyte. Consequently, the voltammetric response (DPV) is dominated by the adsorbed analyte rather than by the redox probe’s charge transfer kinetics alone. In the literature, studies report possible reasons for this profile, including the presence of BC pyrolyzed at temperatures close to 400 °C.^26,37^ According to these studies, pyrolysis temperatures in the order of 400 °C tend to yield carbonaceous materials with disordered carbons resulting from the incomplete depolymerization of lignocellulosic structures, which can partially block electron transfer but simultaneously increase adsorption sites, enhancing analytical current.

### 3.6 Analytical Performance Evaluation

Using the values of previously optimized parameters, DPV experiments were carried out to evaluate the linear range by external calibration, with subsequent steps of adsorbing Tz from 50 to 350 mg L^–1^ in an open circuit, followed by voltammetric scans. As shown in Figure S11, there was a linear correlation between the Tz concentrations and the respective anodic peak currents obtained (*R* ^2^ = 0.993). The LOD and LOQ calculated were 0.17 and 1.57 mg L^–1^, respectively. Additionally, to facilitate a broader comparison, Table 1 also compiles data from previously reported electrochemical methods for Tz determination, enabling a comprehensive evaluation of the analytical performance of the proposed sensor in relation to the literature.

**Table 1:** Analytical Performance Comparison of Electrochemical Sensors for Tz Determination in Different Samples^a^

modified material	electrochemical technique	LOD	sample
graphite on carbon paper^37^	DPV	4.38 × 10^–3^ mg L^–1^	soft drinks
poly(niacin)/CPE^38^	*C–V* and EIS	6.66 × 10^–2^ mg L^–1^	food
BT-I/GCE^39^	DPV and SWV	5.24 × 10^–3^ mg L^–1^	soft drinks
molecularly imprinted polypyrrole/GCE^40^	DPV	5.34 × 10^–4^ mg L^–1^	soft drinks
aminated metal–organic/CPE^41^	DPV	9.46 × 10^–4^ mg L^–1^	soft drinks
poly(glycine)/CPE^42^	DPV	1.51 × 10^–1^ mg L^–1^	candy and soft drinks
GP/NP/SPE^43^	DPV	1.12 mg L^–1^	isotonic, powdered juice, freeze pop, and gelatin
this work	DPV	0.17 mg L^–1^	powdered juice

a Legend: LOD = detection limit; DPV = differential pulse voltammetry; CPE = carbon paste electrode; *C–V* = cyclic voltammetry; EIS = electrochemical impedance spectroscopy; BT-I = bismuthiol I; SWV = square wave voltammetry; GC = glassy carbon electrode; GP = graphite powder; NP = nail polish; and SPE = screen-printed electrode.

In contrast to prior works, this study focuses on the development of a sensor based on low-cost and sustainable materials that are independent of molecular printing techniques or complex nanomaterials. As shown in Table 1, the proposed sensor exhibits an LOD comparable to that of several previously reported electrochemical methods for the determination of Tz. Although some approaches yield lower LOD values, they generally use more complex modifiers, such as nanocomposites, molecularly imprinted polymers, and others, which involve higher costs and more elaborate synthesis steps. Jiang and co-workers^40^ demonstrated the potential of a molecularly imprinted polypyrrole glassy carbon film sensor with an LOD of 0.000534 mg L^–1^ and a linear range of 0.000534 to 0.00534 mg L^–1^. Similarly, a CMPE with aminated metal–organic frameworks showed an LOD of 0.000946 mg L^–1^ in the work of Massah and co-workers.^41^ In contrast, the sensor developed in this work employs biochar obtained from agricultural waste, offering a simpler, lower-cost, and environmentally sustainable alternative while maintaining competitive analytical performance. Additionally, although it offers higher LOD and LOQ, it provides consistent performance in its application, being suitable for the regulatory monitoring of the respective dye in powdered juices.

The method’s accuracy was evaluated by fortifying six samples from two brands with a known concentration of Tz. As demonstrated in Table S2, the method provided recoveries from 84 ± 7.1% to 117 ± 7.3%, which are considered acceptable. It may indicate minimal matrix interference in analytical determination.

Since twilight yellow dye and titanium dioxide can be added to commercially available juices, both were evaluated as interferents in determining Tz. As shown in the voltammograms of Figure S12, twilight yellow and titanium dioxide can interfere with Tz determination. However, based on the recovery obtained in the fortification experiments (data shown in Table S2), the acceptable recovery values did not show a decrease in the Tz analytical signal.

### 3.7 Determination of Tz in Commercially Available Juice Samples

The developed electrode was applied to determine Tz in six samples of commercially available juices from 2 brands. The voltammograms obtained are depicted in Figure 6, and the results are described in Table 2.

**Figure 6:**
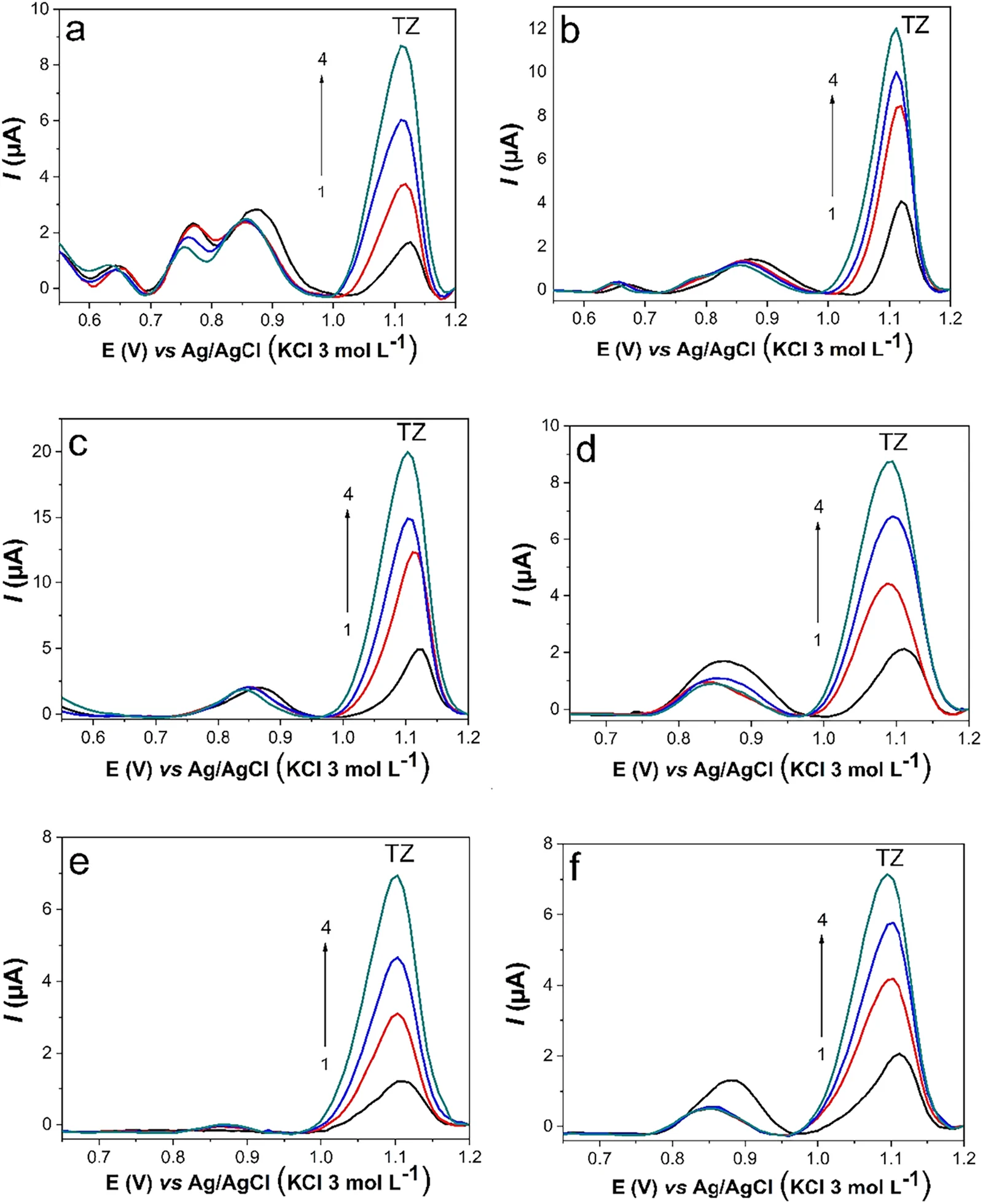
Tz determinations on CMPE-BC28 by DPV in samples: (a) orange flavor, (b) lemon flavor, and (c) passion flavor relative to brand 1 and (d) orange flavor, (e) lemon flavor, and (f) passion flavor relative to brand 2. Experimental conditions: phosphate buffer solution 0.1 mol L^–1^ (pH = 2), *E*_inicial_: 0.5 V; *E*_final_: 1.2 V; amplitude: 0.12 V; *E*_step_: 0.008 V (a: sample, 1–4: calibration from 50 to 150 mg L^–1^ of Tz).

**Table 2:** Concentration Determined and Quantifiable Limits of Tz in Fruit Juice Orange, Lemon, and Passion Flavors (Brands 1 and 2) Using the CMPE-BC28 by DPV

sample	mean Tz concentration determined ± standard deviation (g kg^–1^)	Tz quantifiable limits (g kg^–1^)
brand juice 1		
lemon	2.21 ± 0.01	0.104
orange	0.48 ± 0.01	0.031
passion	1.66 ± 0.05	0.104
brand juice 2		
lemon	0.05 ± 0.01	0.021
orange	0.74 ± 0.03	0.031
Passion	0.55 ± 0.01	0.063

As depicted in Table 2, the Tz concentration is generally highest in samples from brand 1, and among all samples, the Tz concentrations determined were from 0.05 to 2.21 g kg^–1^. Vidotti and Rollemberg^44^ determined Tz in powdered juices with three different flavors by high-performance liquid chromatography (HPLC), and the concentrations quantified varied from 0.148 to 1.212 g kg^–1^, demonstrating the accordance between the results presented in our work. Table 1 also shows the method́s LOQ varied between 0.021 and 0.104 g kg^–1^. These values motivate the proposed method, as EFSA^45^ reports that the maximum allowed concentrations of Tz in foods range from 0.05 to 0.5 g kg^–1^, and in juices from 0 to 100 mg L^–1^.

It is essential to note that the method proposed in this work allows the use of nontoxic reagents, making this electrode development less impactful on the environment. Furthermore, the proposed approach enables the valorization of bioavailable materials such as rice husks, which are commonly considered agricultural waste.

## 4 Conclusion

It was possible to successfully evaluate the potential of rice husk BC to modify CMPE. The experimental planning by CCRD allowed the acquisition of a combination of pyrolysis parameters that provided an excellent adsorption capacity and electroanalytical response. Optimizing the graphite composite with rice husk BC improved the sensitivity and indicated possible analytical applications. In this way, an electroanalytical method was proposed that offers environmental appeal and aligns with the principles of green analytical chemistry, enabling the addition of technological value to rice husks, an agricultural residue in need of alternative destinations. In addition, the results obtained provide a basis for applying rice husk BC in new experiments to explore its adsorption capacity and electroanalytical response to food dyes. Despite the promising results, some limitations should be considered, including the higher detection limits compared with more sophisticated sensors and the evaluation restricted to a limited number of samples. Future studies should focus on improving sensitivity and expanding the application to different food matrices and potential interferents.

## Supplementary Material



## Data Availability

Data used are available throughout the manuscript text.

## Notes

CCRD experimental design matrix (Table S1), response surface plots obtained from the optimization study (Figures S1–S6), optimization of the voltammetric parameters (Figures S7–S9), cyclic voltammograms at different scan rates (Figure S10), analytical curve (Figure S11), recovery results for fruit juice samples (Table S2), and interference studies (Figure S12) (PDF)
