# Supplementary material for: Rice Husk Biochar: A Promising Material for the Development of Electrochemical Devices for Tartrazine Determination
Source: ACS Omega. 2026 Aug 10;11(33):48929–38. doi: 10.1021/acsomega.5c12503 (PMC13520528; doi:10.1021/acsomega.5c12503)
Supplement: Supplementary file 1 [file ao5c12503_si_002.pdf]

Rice husk biochar: a promising biomaterial for developing devices applied to tartrazine determination

Leandro Silva de Almeida[a], Ana Paula de Oliveira Lopes Antunes[a], Eliézer Quadro Oreste[a], Mery Luiza Garcia Vieira[b], Luiz Antonio de Almeida Pinto[b], Tito Roberto Sant'Anna Cadaval Junior[b], Daiane Dias[a]*

[a] Laboratório de Eletroespectro Analítica (LEEA), School of Chemistry and Food Federal University of Rio Grande, 96203-900, Rio Grande, Brazil

[b] Laboratório de Tecnologia Industrial, School of Chemistry and Food Federal University of Rio Grande, 96203-900, Rio Grande, Brazil

*Corresponding author: daianezd@gmail.com

Table S1. Design matrix and current responses of voltammetric analyses for tartrazine dye according to rotational central composite design

Pyrolysis	Temperature (°C)	Mass (g)	Heating rate (°C min^{-1})	Time (min)	Sensor	Current (µA)
1	300	20	10	90	CMPE-BC1	0.32
2	500	20	10	90	CMPE-BC2	0.27
3	300	40	10	90	CMPE-BC3	0.34
4	500	40	10	90	CMPE-BC4	0.40
5	300	20	20	90	CMPE-BC5	0.29
6	500	20	20	90	CMPE-BC6	0.29
7	300	40	20	90	CMPE-BC7	0.23
8	500	40	20	90	CMPE-BC8	0.26
9	300	20	10	150	CMPE-BC9	0.31
10	500	20	10	150	CMPE-BC10	0.32

11	300	40	10	150	CMPE-BC11	0.32
12	500	40	10	150	CMPE-BC12	0.31
13	300	20	20	150	CMPE-BC13	0.33
14	500	20	20	150	CMPE-BC14	0.35
15	300	40	20	150	CMPE-BC15	0.45
16	500	40	20	150	CMPE-BC16	0.27
17	200	30	15	120	CMPE-BC17	0.16
18	600	30	15	120	CMPE-BC18	0.36
19	400	10	15	120	CMPE-BC19	0.32
20	400	50	15	120	CMPE-BC20	0.30
21	400	30	5	120	CMPE-BC21	0.28
22	400	30	25	120	CMPE-BC22	0.41
23	400	30	15	60	CMPE-BC23	0.34
24	400	30	15	180	CMPE-BC24	0.39
25	400	30	15	120	CMPE-BC25	0.33
26	400	30	15	120	CMPE-BC26	0.33
27	400	30	15	120	CMPE-BC27	0.32

14

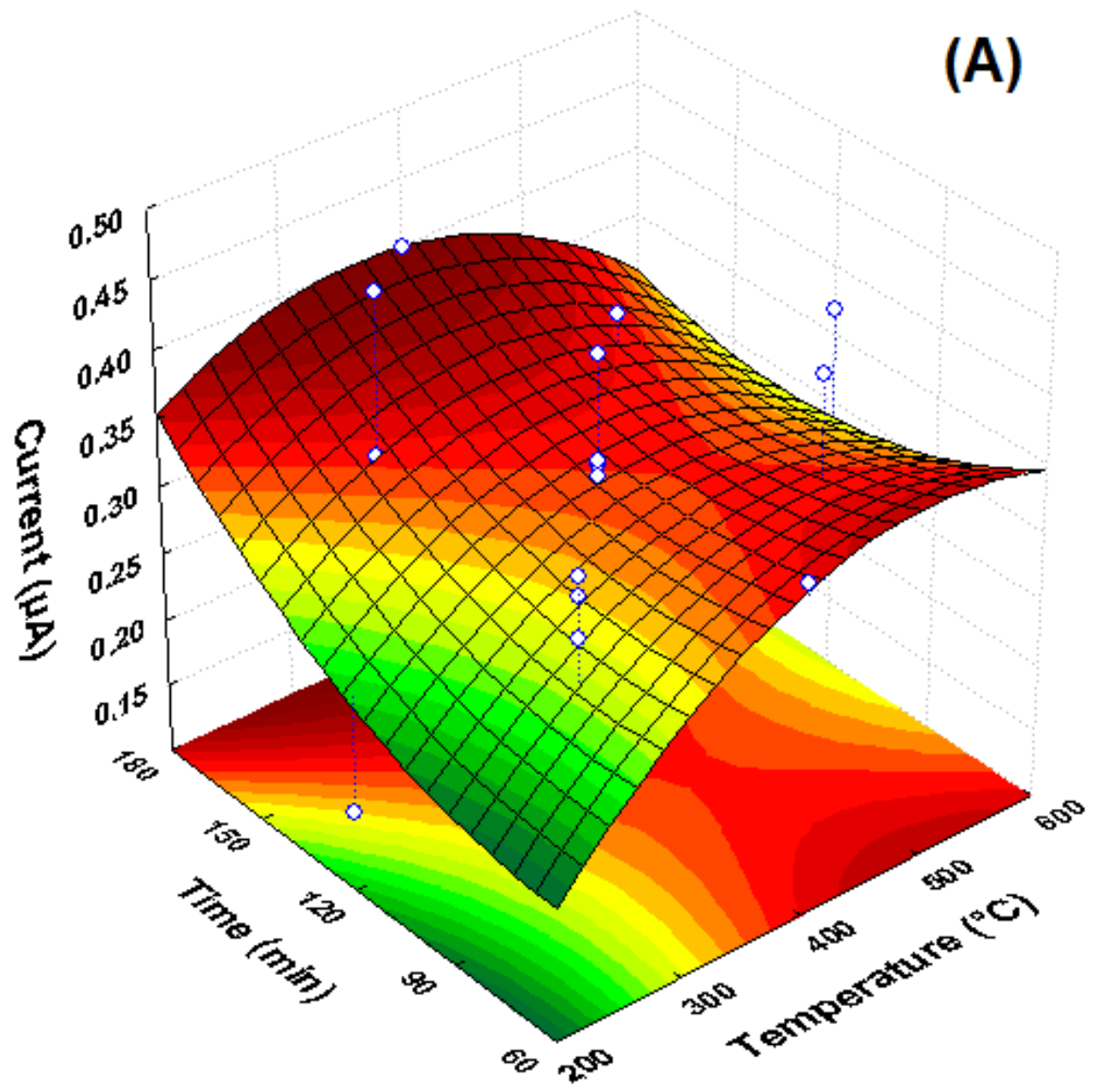

Figure S1. Response surface graph for variation of the time and temperature obtained by CCRD.

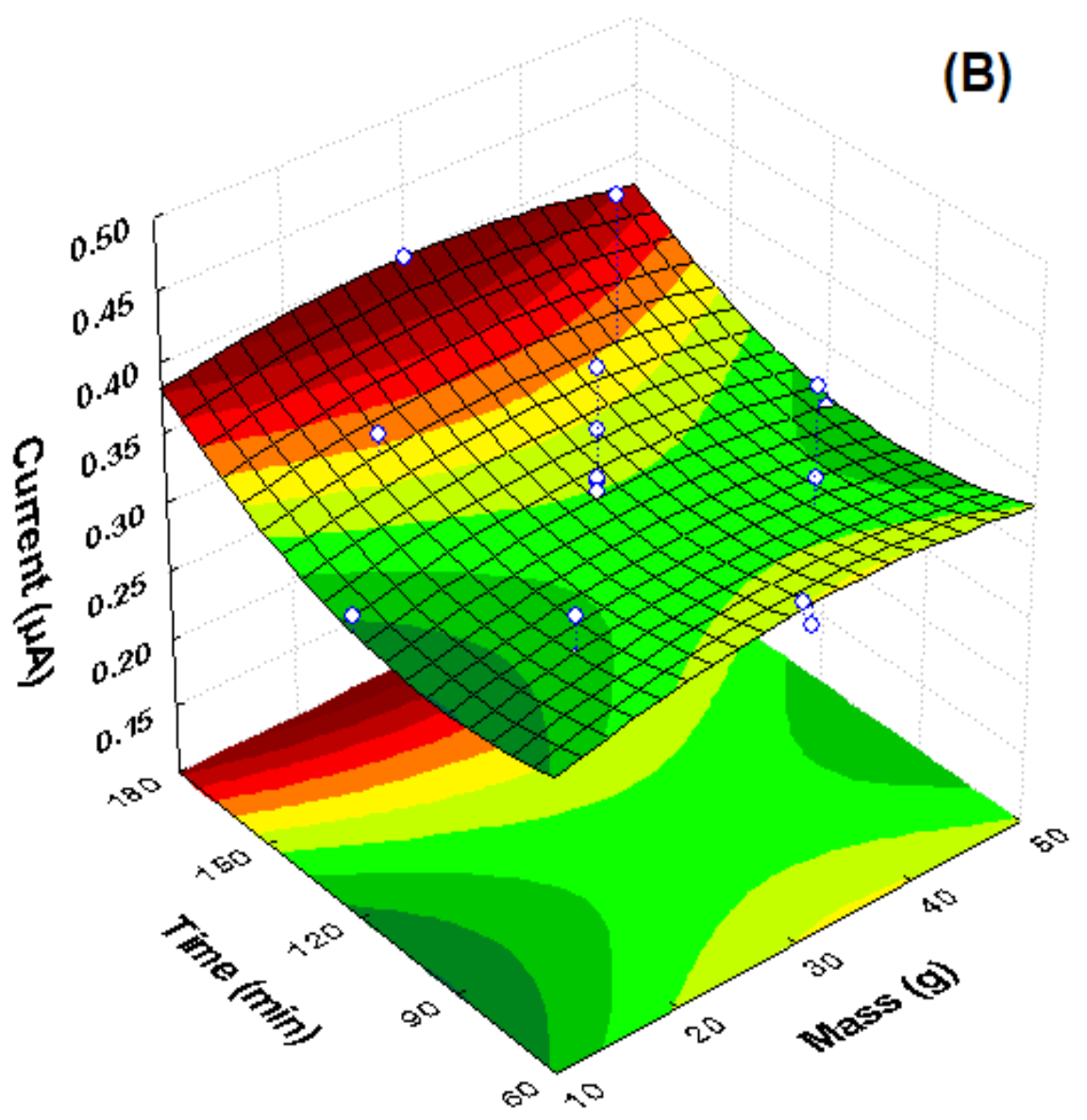

Figure S2. Response surface graph for variation of the time and mass obtained by CCRD.

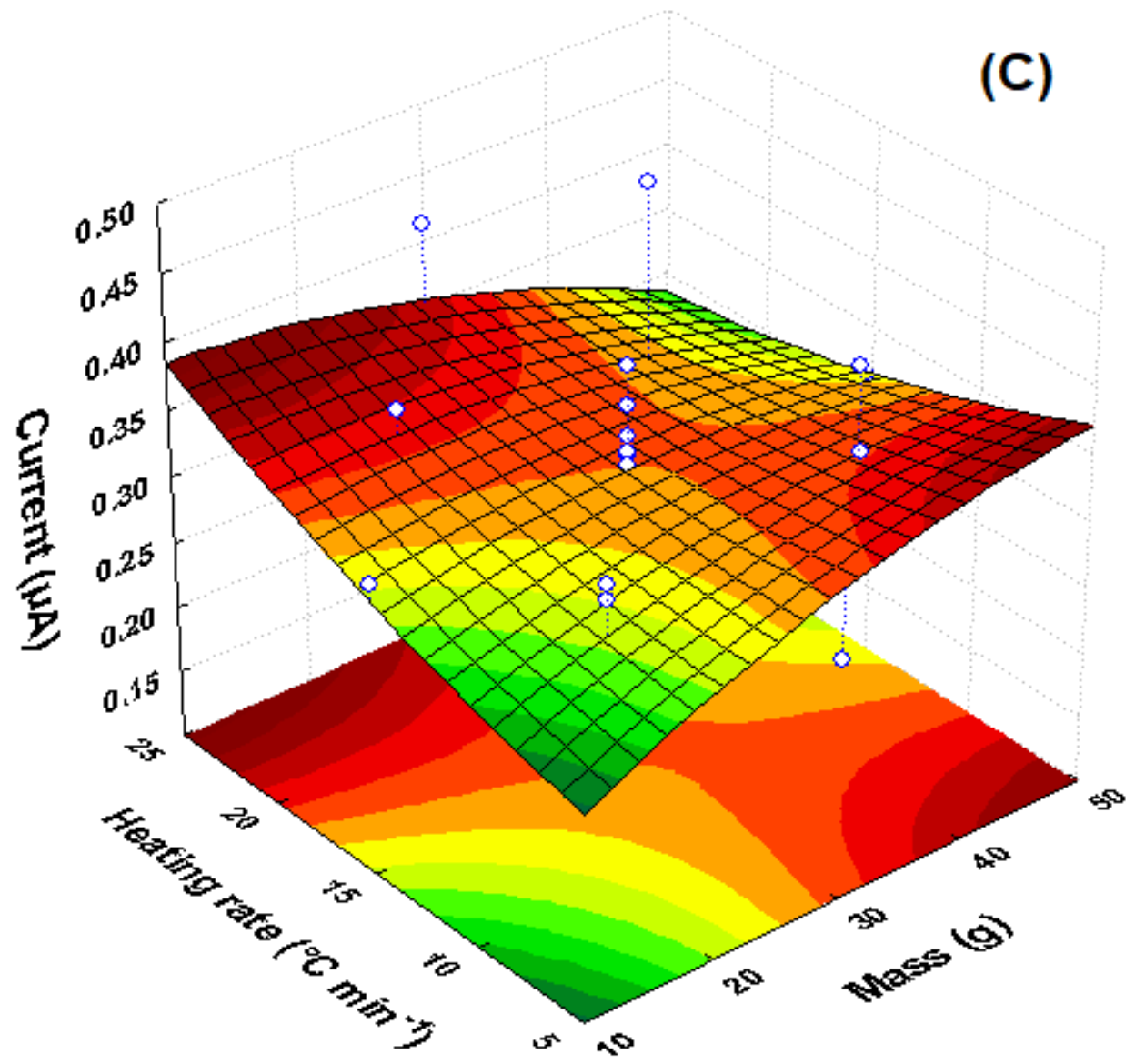

Figure S3. Response surface graph for variation of the heating rate and mass obtained by CCRD.

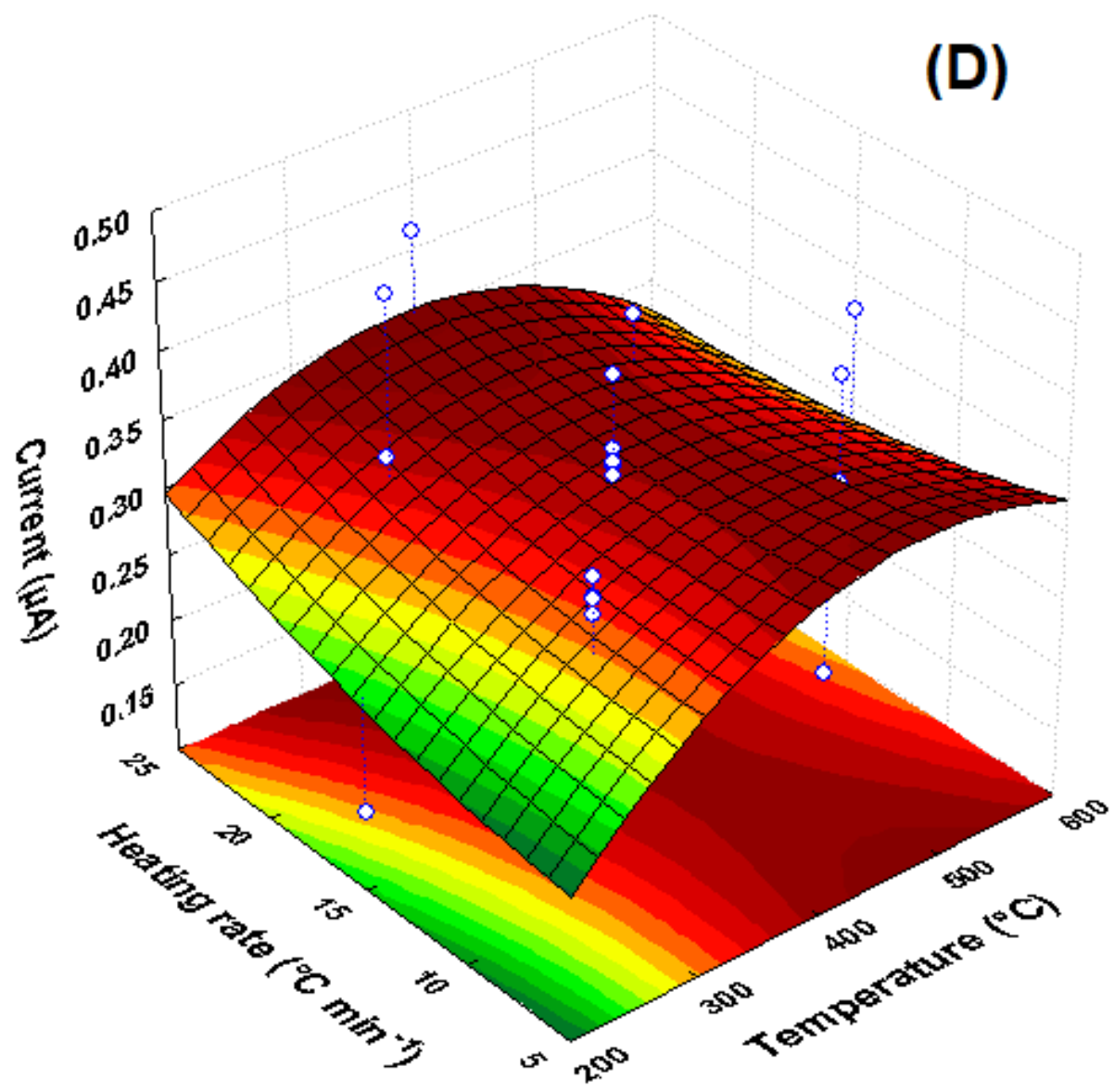

Figure S4. Response surface graph for variation of the heating rate and temperature obtained by CCRD.

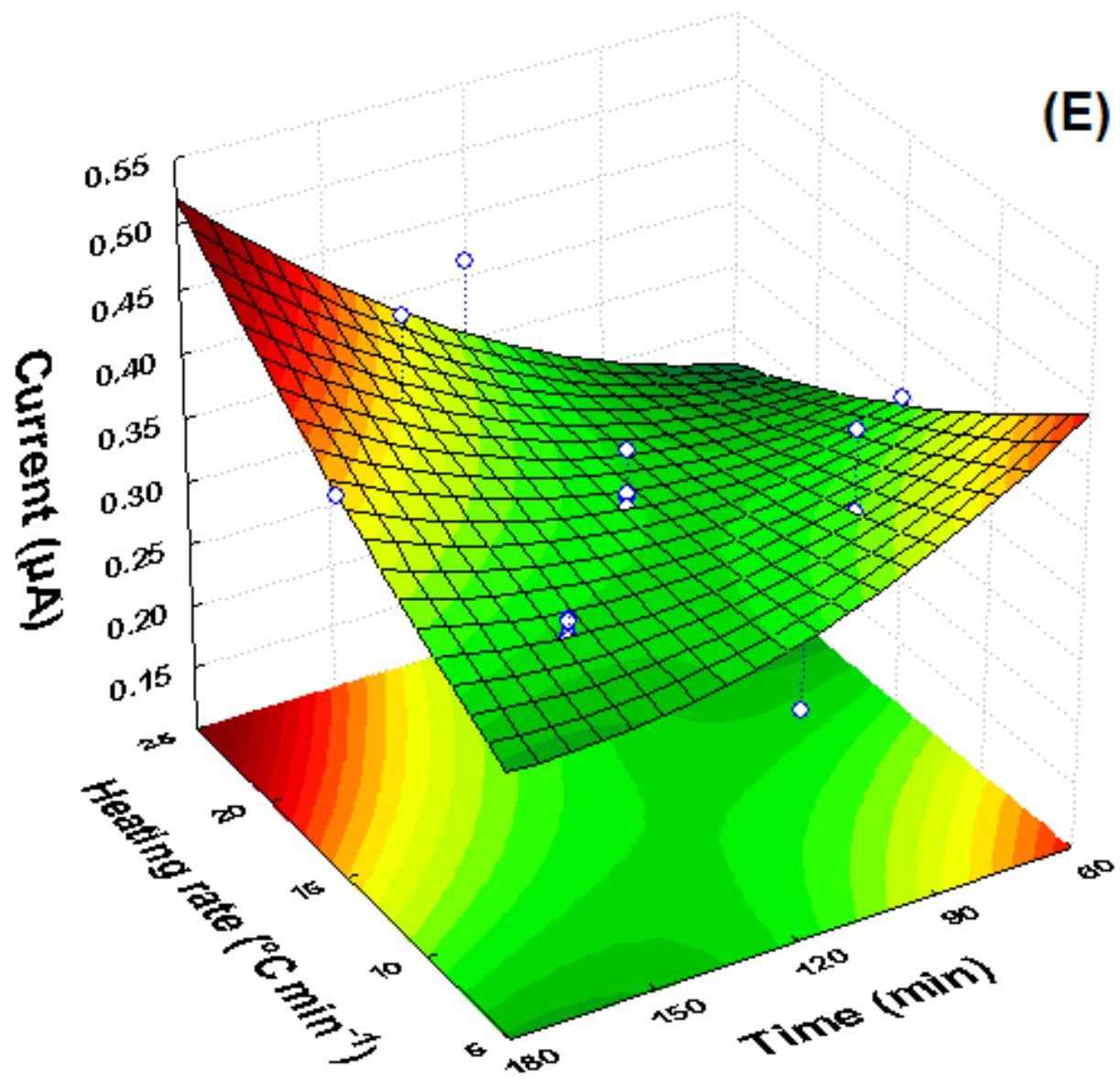

Figure S5. Response surface graph for variation of the heating rate and time obtained by CCRD.

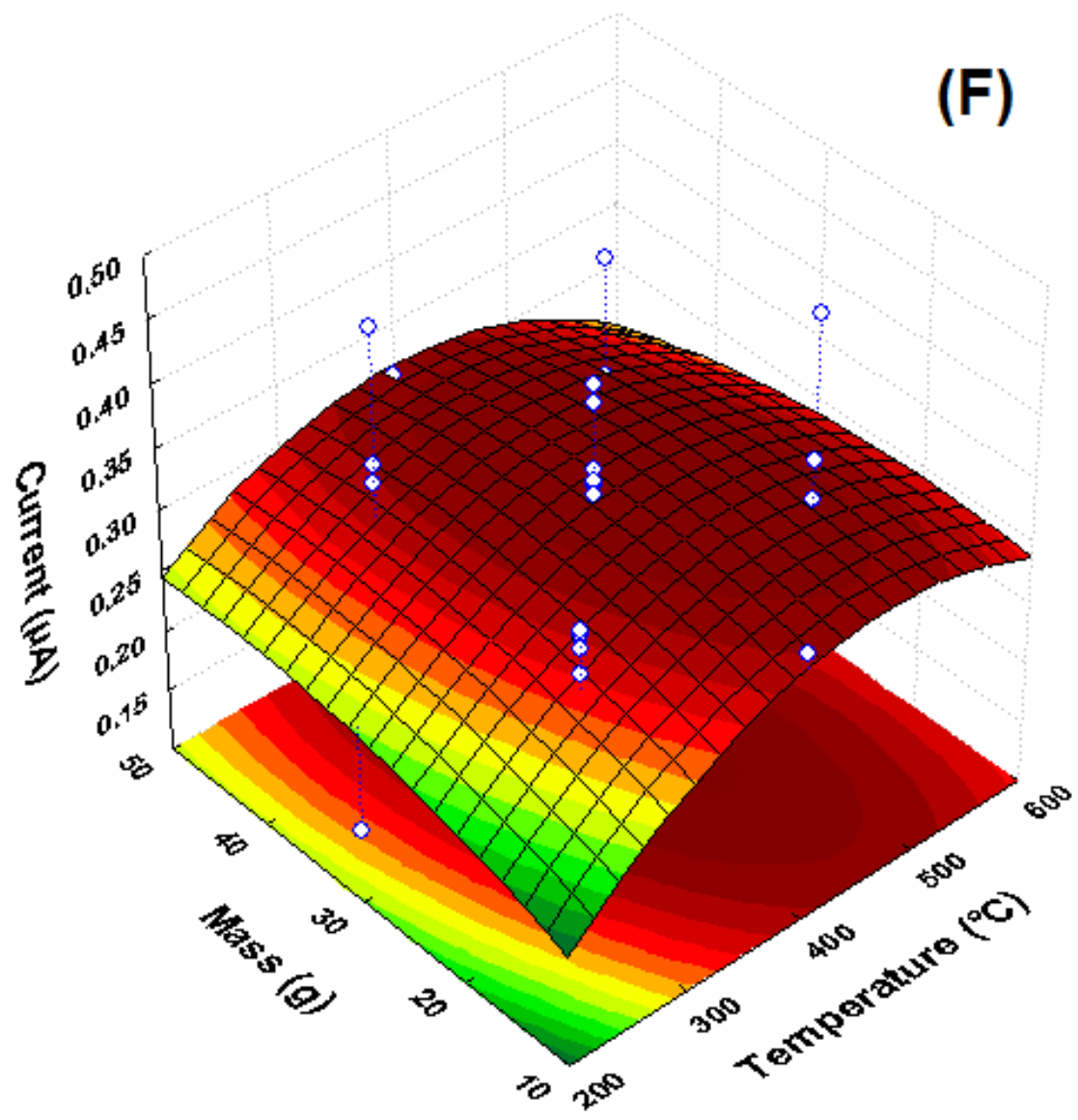

Figure S6. Response surface graph for variation of the mass and temperature obtained by CCRD.

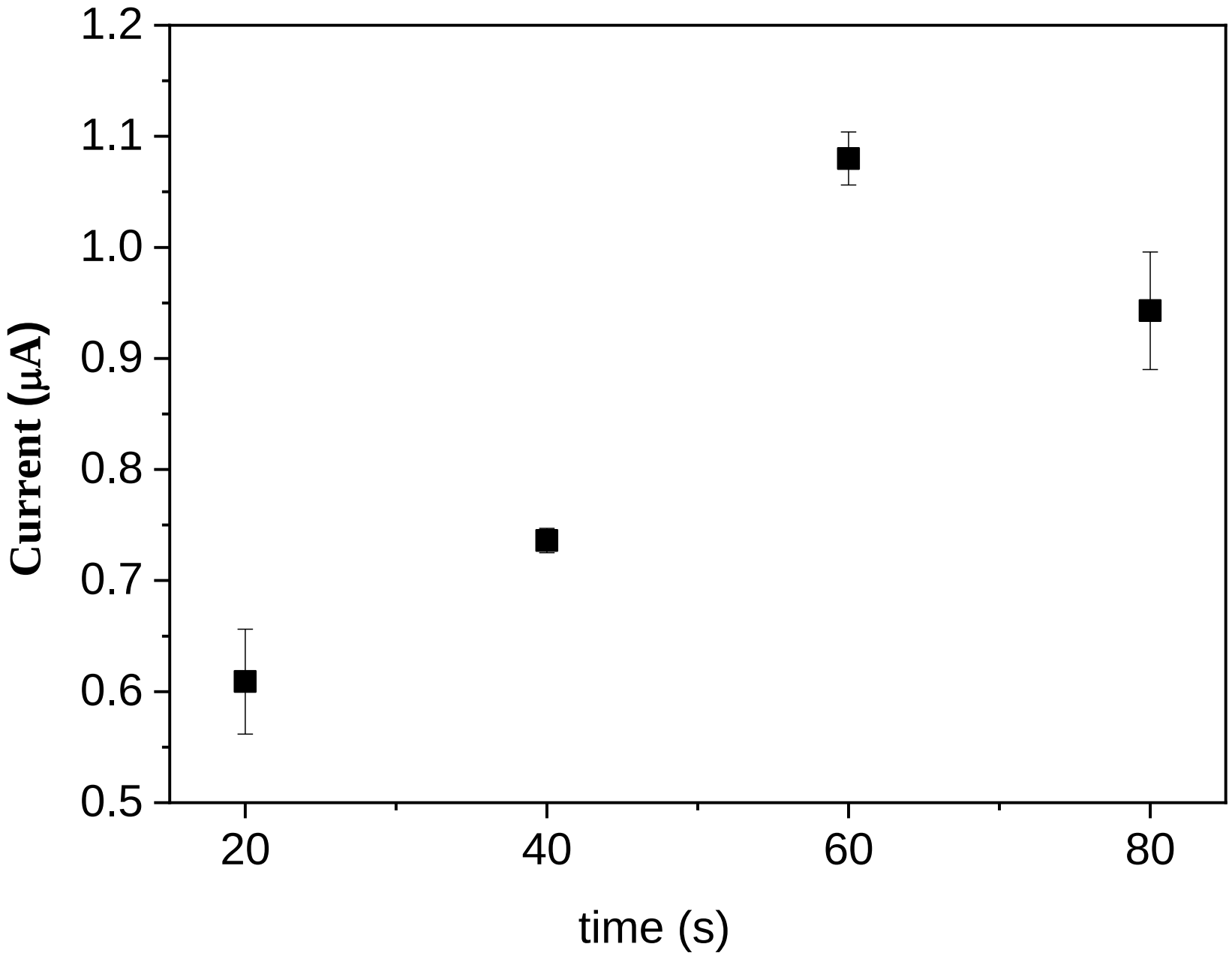

Figure S7. Influence evaluation of the adsorption time (from 20 to 80 s) on the current responses of tartrazine (open circuit adsorption of tartrazine 0.15 g L^{-1} during 60 s) in the CMPE-BC28 by DPV. Experimental conditions: phosphate electrolyte 0.1 mol L^{-1}, pH 2.0, $E_{inicial}$: 0.5 V, E_{final}: 1.2 V, E_{step}: 0.003 V, amplitude: 20 mV)

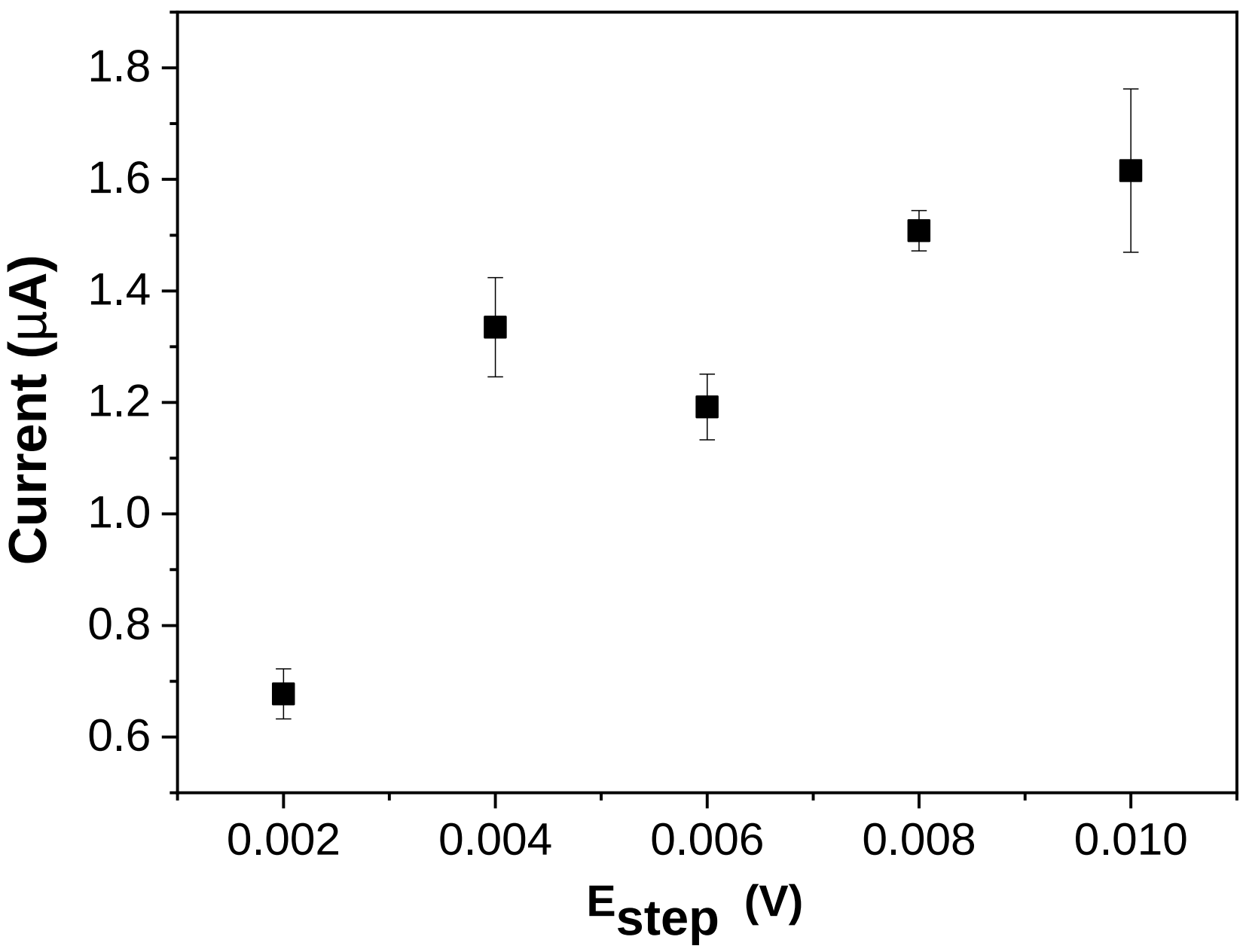

Figure S8. Influence evaluation of the E_{step} (from 0.002 to 0.01 V) on the current responses of tartrazine (open circuit adsorption of tartrazine 0.15 g L^{-1} during 60 s) in the CMPE-BC28 by DPV. Experimental conditions: phosphate electrolyte 0.1 mol L^{-1}, pH 2.0, $E_{inicial}$: 0.5 V, E_{final}: 1.2 V, E_{step}: 0.003 V, amplitude: 20 mV).

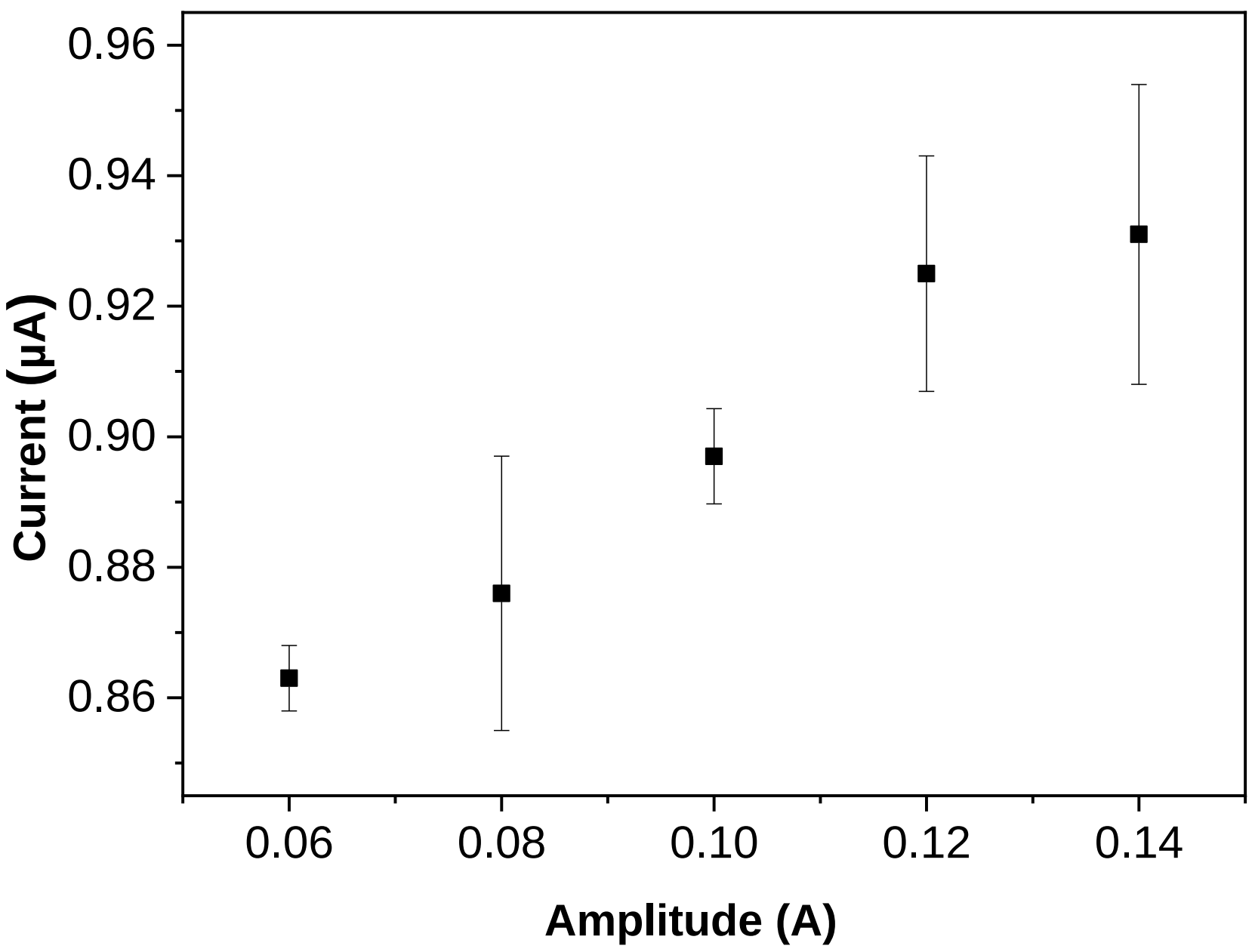

Figure S9. Influence evaluation of the amplitude (from 0.06 to 0.14 V) on the current responses of tartrazine (open circuit adsorption of tartrazine 0.15 g L^{-1} during 60 s) in the CMPE-BC28 by DPV. Experimental conditions: phosphate electrolyte 0.1 mol L^{-1}, pH 2.0, $E_{inicial}$: 0.5 V, E_{final}: 1.2 V, E_{step}: 0.003 V, amplitude: 20 mV).

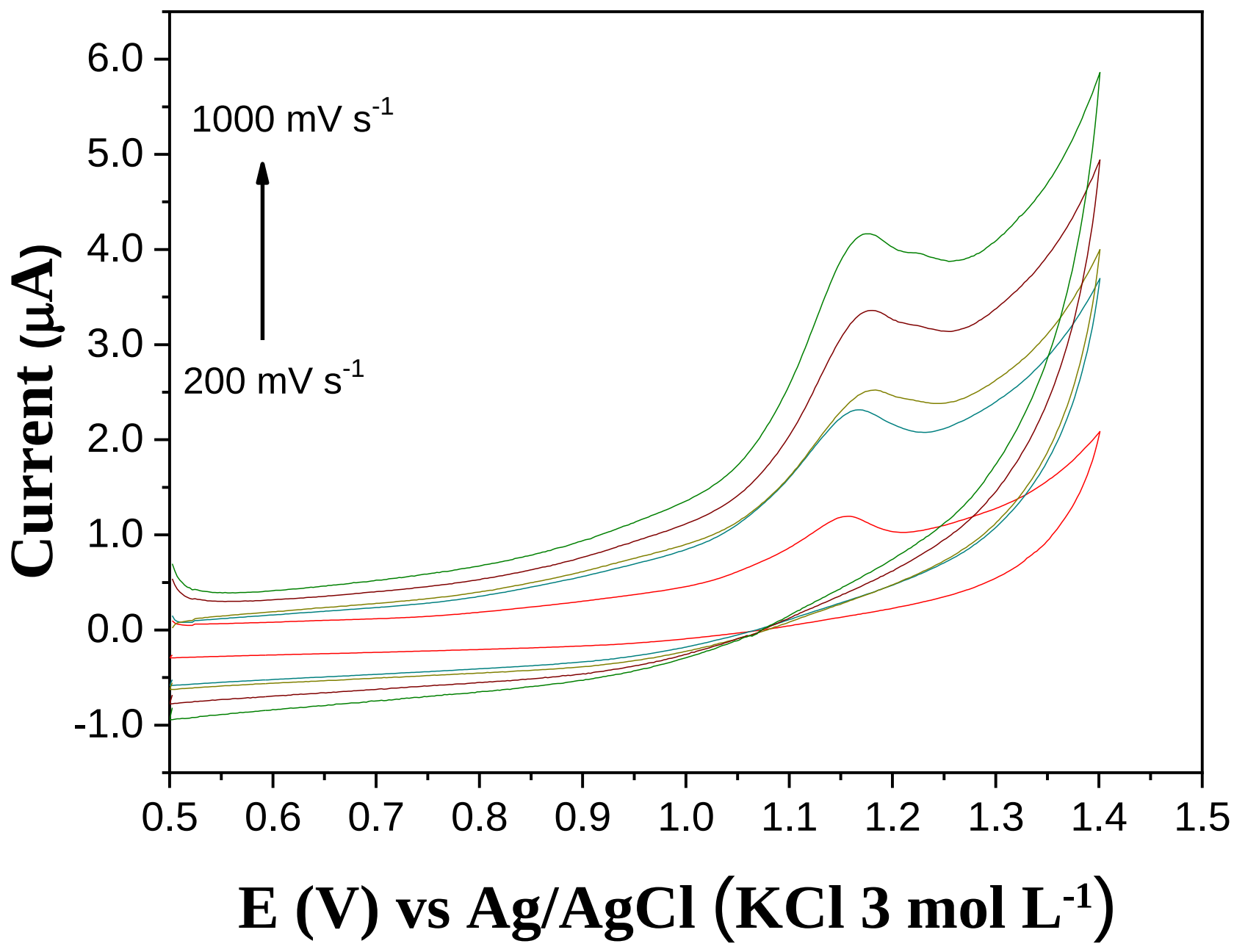

Figure S10. Cyclic tartrazine voltammograms on CMPE-BC28, for scan speeds ranging from 0.1 to 1.0 V s^{-1}. Experimental conditions: open circuit tartrazine adsorption (0.5 g L^{-1}) and in the voltammetric cell, phosphoric acid 0.1 mol L^{-1}, pH 2.0, $E_{initial}$: 0.5 V and E_{final}: 1.4V.

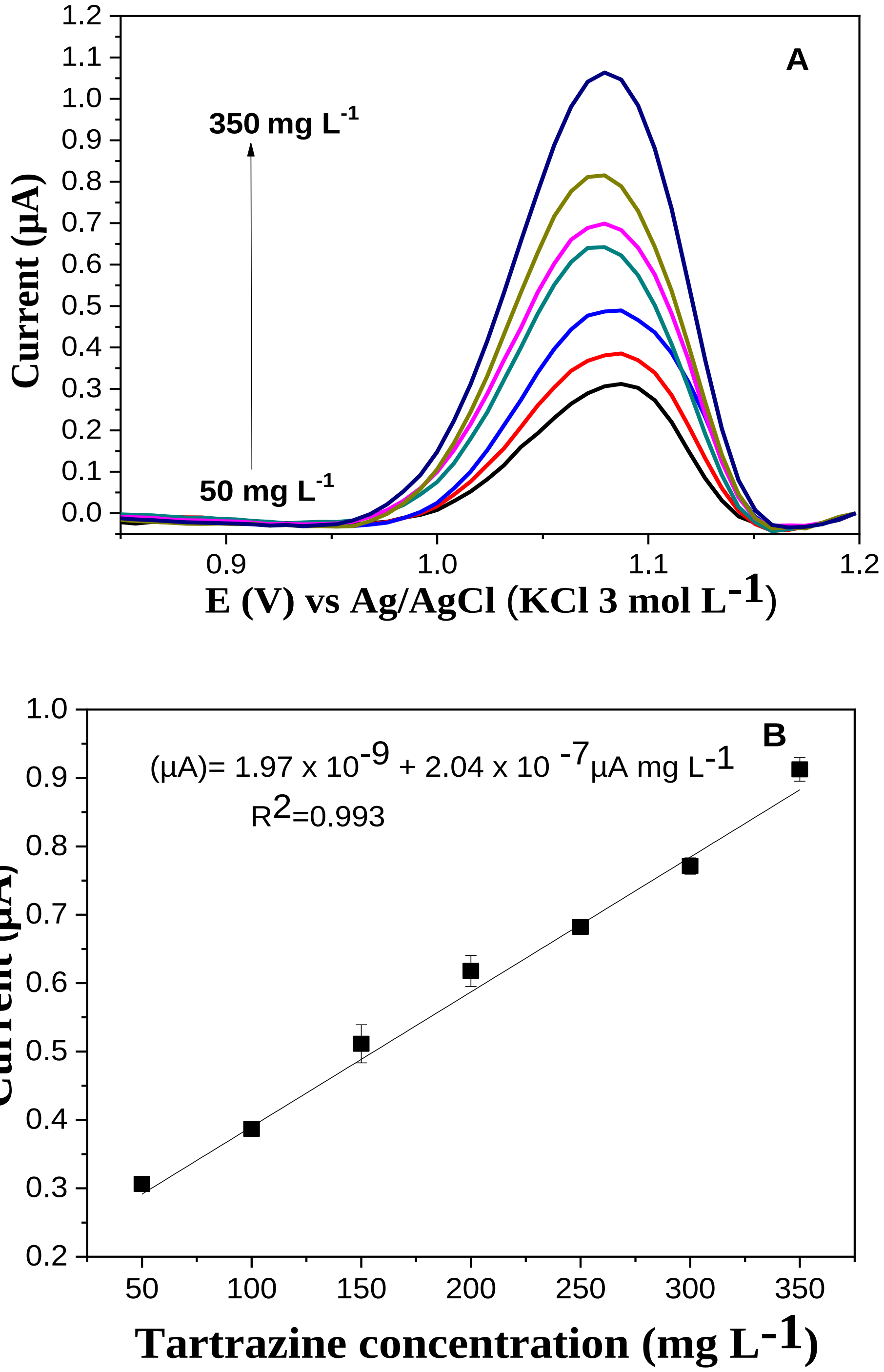

Figure S11. Tartrazine voltammograms (50 a 350 mg L^{-1}) by DPV (a) and analytical curve (b). Experimental conditions: phosphate buffer 0.1 mol L^{-1} (pH 2.0), $E_{initial}$: 0.5 V; E_{final}: 1.5 V, amplitude: 0.12V, E_{step}: 0.008V.

Table S2. Addition and recovery of tartrazine in fruit juice orange, lemon and passion flavor (brands 1 and 2), using the CMPE-BC28 in the sample spontaneous adsorption step. Followed by the determination by DPV to being: ($E_{initial}$: 0.5 V; E_{final}: 1.5 V; amplitude: 0.12 V; E_{step}: 0.008 V)

Sample	Fortified Concentration (g L^{-1})	Determined Concentration (g L^{-1})	Recovery (%)
Brands 1			
Lemon	0.035	0.041 ± 0.003	117 ± 7,3
Orange	0.100	0.084 ± 0.006	84 ± 7,1
Passion	0.050	0.056 ± 0.002	112 ± 3,6
Brands 2			
Lemon	0.010	0.010 ± 0.001	100 ± 10,0
Orange	0.100	0.115 ± 0.006	115 ± 5,2
Passion	0.025	0.025 ± 0.002	100 ± 8,0

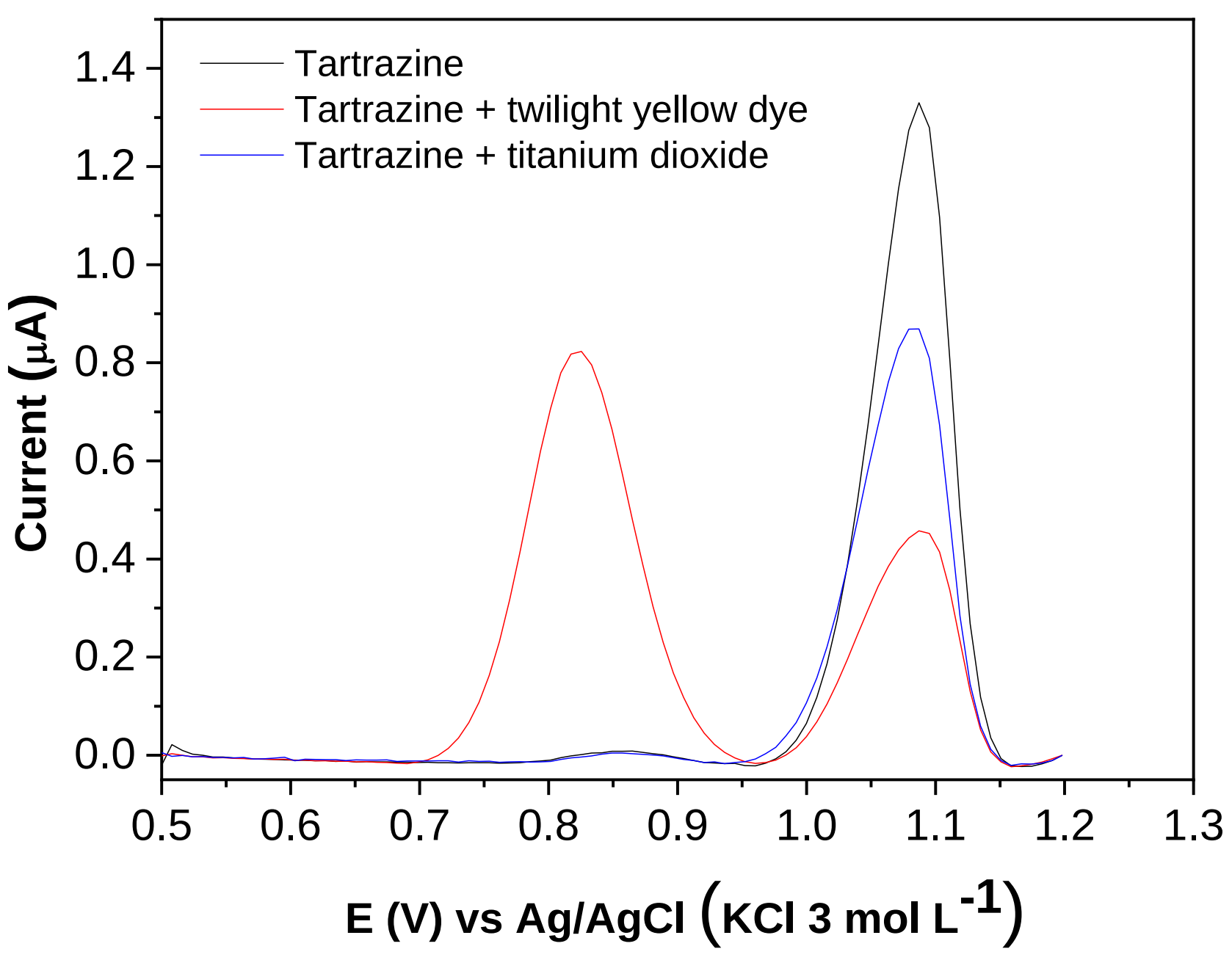

Figure S12. Voltammograms by DPV of tartrazine (150 mg L^{-1}), tartrazine containing twilight yellow (300 mg L^{-1}) and tartrazine containing titanium dioxide (300 mg L^{-1}). Experimental conditions: phosphate buffer 0.1 mol L^{-1} (pH 2.0), $E_{initial}$: 0.5 V; E_{final}: 1.5 V, amplitude: 0.12V, E_{step}: 0.008V.
